# Resilience as a predictive factor towards a healthy adjustment to grief after the loss of a child to cancer

**DOI:** 10.1371/journal.pone.0214138

**Published:** 2019-03-21

**Authors:** Hilde Kristin Vegsund, Trude Reinfjell, Unni Karin Moksnes, Alexandra Eilegård Wallin, Odin Hjemdal, Mary-Elizabeth Bradley Eilertsen

**Affiliations:** 1 Department of Public Health and Nursing, Centre for Health Promotion Research, NTNU Norwegian University of Science and Technology, Trondheim, Norway; 2 Department of Psychology, NTNU Norwegian University of Science and Technology, Trondheim, Norway; 3 School of Education, Health and Social Studies, Dalarna University, Falun, Sweden; Harvard TH Chan School of Public Health, UNITED STATES

## Abstract

**Introduction:**

Grief among bereaved parents is known to cause psychological distress and physical illness, but knowledge concerning factors that can contribute to health promotion after bereavement is scarce. Childhood cancer remains the most common non-accidental cause of death among children in Norway. The aim of the present study was to explore if resilience factors among cancer-bereaved parents could predict whether they will be able to come to terms with their grief 2–8 years following the loss.

**Methods:**

A Norwegian cross-sectional national survey was conducted among 161 cancer-bereaved parents using a study-specific questionnaire. Logistic regression was used to explore whether resilience factors predicted parents’ grief outcome 2–8 years after their loss.

**Results:**

On the Resilience Scale for Adults (RSA), three of the resilience factors contributed significantly in predicting whether the parents in the present study would come to terms with their grief 2–8 years after the loss their child: “*Perception of self* “(OR 2.08, *p* = .048), *“Social resources”* (OR 2.83, *p* = .008) and *“Family cohesion”* (OR .41, *p* = .025). The results showed a negative relationship between time since loss (2–6 years) and whether the parents answered that they had come to terms with their grief (*p* = < .05). The loss of a parent (OR .30, *p* = .030) combined with the loss of their child had a negative and significant effect on whether they indicated that they had processed their grief.

**Conclusion:**

The total score of RSA and three of the six resilient factors contributed significantly in predicting whether cancer-bereaved parents in the present study indicated that they had come to terms with their grief to a great extent. The present study supports hypotheses that regard resilience as an important contribution in predicting healthy outcomes in people exposed to adverse life events.

## Introduction

In Norway, approximately 200 children under the age of 17 are diagnosed with cancer each year, and the mortality rate of childhood cancer is approximately 20% when including all types of cancer [1]. Losing a child to cancer interrupts the “normal” course of life, as parents are not meant to survive their children, and a child’s death will affect the parents during the course of their lives [2]. The loss of a child is known to cause physical and emotional stress on parents [2]. Studies have shown that parents are at risk of developing psychological distress such as anxiety and depression [3], as well as having an increased risk of hospitalization [4] and mortality due to illness [5]. Bereavement is a universal phenomenon, but a parent’s grief reaction is highly personal. A child’s death is known to cause the most intense and persistent grief in parents [6,7], and mothers are known to have more intense grief reactions than fathers [8]. In one study [9], it was found that the majority of bereaved parents indicated that grief was a lifelong process. However, most parents appear to have a healthy adjustment to the loss of their child [10], and previous research has shown that grief reaction is greatly associated with the amount of time that has passed since the loss [11,12].

The Dual Process Model of Bereavement (DPM) has contributed to a stronger theoretical foundation in the understanding of grief reactions [13]. The DPM divides bereavement phenomena into loss- and restoration-oriented stressors and includes the process of dynamic and regulatory oscillation, where bereaved individuals intermittently confront and avoid the various aspects of the grieving process [14,15]. Loss orientation refers to how individuals cope with the loss experience regarding the deceased child [15]. Restoration-orientation refers to secondary stressors related to the loss, such as bereaved parents will go through an adjustment from being a parent to being the “parent of a deceased child” [15]. Contrary to previous grief theories, the DPM includes the interaction between environmental factors and the individual grief work to understand the concept of grief [13].

### Resilience

After decades focusing on pathology and the treatment of illness following adversities or stressful life events, the human capacity to adapt to adversities has been neglected [16]. Currently, the focus has shifted to include factors that can contribute to the promotion of health after bereavement [17]. Resilience is one of many concepts covered under health promoting constructs [18], and can be viewed as a buffer with a healthy outcome following exposure to risk [19], whereas vulnerability is associated with the likelihood of a negative outcome when exposed to risk [20].

Researchers have tried to define the construct of resilience, but there is no consensus of a common definition [21]. Nevertheless, several researchers have agreed that the three most important predictors and determinants associated with resilience are: *1) psychological individual traits* (e.g., self-efficacy, coping skills, optimism and empathy), *2) affectional bonds within the family*, and *3) a support system outside the family* (e.g., friends, colleagues and neighbors) [20,22–25]. Resilient individuals have been found to have successful adaptation to stressful life events, including the loss of a person close to them [26]. Recent research indicates that some individuals show a consistently low level of, or an absence of, grief reactions after the loss of a person close to them [2]. Individuals who are able to function close to normal in the face of adverse life events are referred to as resilient human beings [22]. Some central themes within resilience research are the question of why some individuals seem to resist adverse events without developing physical or psychological ill health [21] and why resilient individuals are able to recover more readily than others after a painful loss [27]. Resilience traits within individuals, as well as social support from family and other networks can increase the likelihood of a positive adaptation to adverse life events [27], and parents who possess protective factors have a greater chance of a healthy outcome following the loss of a child [28]. A definition of resilience is “the protective factors, processes, and mechanisms that contribute to a good outcome despite experiences with stressors shown to carry significant risk for developing psychopathology” [19]. A factor can be either a risk or an asset, depending on the level of exposure to it, and the nature of the factor [20]. Research on resilience factors among cancer-bereaved parents is scarce. To the authors’ knowledge, there is little research concerning resilience as a predictor of grief outcome of parents after the loss of a child to cancer.

### Aim

The aim of the present study was to explore whether resilience factors among cancer-bereaved parents were associated with their grief outcome 2–8 years following the loss. We hypothesized that; parents who had a positive high score on the resilient scale for adults (RSA) would come to terms with their grief to a greater degree compared with parents who had a lower score on the RSA.

Further, time since the loss would predict to which degree the parents would indicate that they have come to terms with their grief. The parents who lost their child 8 years earlier were expected to have come to terms with their grief to a greater extent compared with the parents who lost their child 2 years previously.

In addition, we further examined whether parents’ adjustment to the loss of their child would be associated with the following factors: the loss of another child, the loss of a parent or the loss of a parent-in-law.

## Materials and methods

### Procedure

The data in the present study are from a Norwegian national study conducted among bereaved parents who lost their child to cancer 2–8 years earlier. A total number of 246 children, who died from cancer before the age of 24 in the period from January 2009 to December 2014 were identified through the Cancer Registry of Norway and the Norwegian Cause of Death Registry. The Norwegian National Population Register identified the 474 parents. The inclusion criteria for participation in the study were that the parents had to live in Norway and speak and write Norwegian. An invitation letter, including a note to give their written consent was sent to all 474 parents who met the inclusion criteria in June 2017. After the invitation letter was distributed, parents of five children contacted the research team and informed us that their child did not die from cancer but of other causes; hence ten parents were removed from our lists. A reminder was distributed in July 2017 to the 264 parents who had not responded to the first request. Initially, a total of 230 (49,6%) parents gave their written consent to participate in the study. Of the 230 parents who gave their consent, eleven parents (4%) withdrew their written consent, nine of them indicated that it was too emotionally demanding to complete the questionnaire, and two gave no reason for the withdrawal. Fifty-eight (25.2%) parents who had given consent to participation did not return the questionnaire. Altogether, 161 parents returned the questionnaire, yielding a response rate of 34.8%. The questionnaire was distributed in July and August 2017 to all parents who had returned a written consent. The data collection was completed in the beginning of October 2017.

### The questionnaire

The questionnaire is a translated version of a Swedish study-specific questionnaire for cancer-bereaved parents [29]. The questionnaire was translated into Norwegian for the purpose of the present study. Two Norwegians well-acquainted with the Swedish language translated the questionnaire into Norwegian. Two bilingual native Swedish persons who have lived in Norway for several years and speak both Norwegian and Swedish fluently made the back translations. Six interviews were conducted with bereaved parents to ensure that the questions and response alternatives were understood as intended and were appropriate for use among the Norwegian population of cancer-bereaved parents.

### Measures

*Grief* as the dependent variable was measured with the question; “Do you think you have come to terms with your grief?” There were three response options: “No, not at all” (2.5%), “Yes, a little” (60.8%), and “Yes, fairly much” (36.7%). There were three missing responses for that question. The dependent variable had a severely skewed distribution. Only four parents answered on the first response alternative “No, not at all”; hence, the two first response categories were merged.

### Resilience scale

The resilience scale for adults (RSA) developed by Friborg and Hjemdal [22], is validated in several countries [30–32] and is frequently used and validated in a Norwegian population [19,22,33–35]. The items are measured with a seven-point semantic differential scale, with 17 negatively phrased statements and 16 positively phrased statements [35]. The negative statements are reversed before statistical analyses. The higher the score on RSA scale, the more protective factors an individual is assumed to have, e.g., better psychological health, and thus, the individual seems to cope better with adverse life events.

RSA consists of six factors divided into 1) *intrapersonal resources* (*“Perception of self”*, *“Planned future”*, *“Social competence”* and *“Structured style”*), and 2) *interpersonal resources* (*“Family cohesion”* and *“Social resources”*) [34]. The first factor, *“Perception of self”*, consists of six items regarding self-confidence, the ability to solve problems and trust in the decisions made. Factor two, *“Planned future”*, entails four items regarding a person’s view of whether or not they have an optimistic view of the future. The third factor, *“Social competence”*, consists of six items, and includes statements regarding flexibility, friendships, and how individuals see themselves in social settings. The fourth factor, *“Structured style”*, consists of four items regarding whether the individual is good at planning their time or has clear goals for the future. *“Family cohesion”* is the fifth factor and contains six items including statements about supportiveness and views of what is important in life among the family members. The last factor, *“Social resources”*, has seven items concerning the individual’s perception of support in their life, and who can help in difficult times.

*Time since loss* is measured in years. Parents who lost their child two to eight years before were included in the study.

### Control variables

The control variables included in the study are sex, marital status, loss of another child, loss of a parent and loss of a parent-in-law (Table 1).

**Table 1:** Characteristics of participants.

Demographics	n	%
Loss of a boy	73	45.34
Loss of a girl	88	54.66
**Age**^a^ (years), median ±SD^b^	161	53 ±7.59
**Sex**		
Men	63	39.13%
Women	98	60.87%
**Marital status**		
Married to the deceased child’s other parent	113	70.19%
Married/living with another partner than the deceased child’s other parent	16	9.94%
Living alone but have a partner	13	8.07%
Single	19	11.80%
**Education**		
Primary and lower secondary school	6	3.77%
High school	25	15.72%
Technical college	32	20.13%
College/university (3 years)	45	28.30%
College/university (4 years or more)	51	32.08%

Note:

^a^ Parents age answering the questionnaire

^b^ SD = standard deviation.

The sex variable is coded with men as the reference variable. Marital status is coded with “parents who are still married to the deceased child’s other parent” as 0, and “parents living with or married to another partner than the deceased child’s other parent,” “having a partner but not living together,” and “single” as 1.

### Analysis

Data were analyzed with STATA Statistics/Data analysis version 15.1 (College Station Texas 77845, StataCorp LLC, USA). Descriptive statistics were conducted for control variables (Table 1), and Pearson’s correlations analysis and Cronbach’s alphas were used for the resilience scale (Table 2). Logistic regression was performed to estimate the probability that bereaved parents had worked through their grief. The model included the six resilience factors, time since loss of their child measured in years, and control variables.

**Table 2:** Means, standard deviations, Cronbach’s alphas and Pearson’s correlations for the RSA scale.

	Mean	SD	*α*	PS	PF	SC	FC	SR	SS
PS	5.07	1.17	.86	-					
PF	4.93	1.35	.86	0.69*	-				
SC	4.74	1.16	.80	0.51*	0.33*	-			
FC	5.41	1.15	.87	0.30*	0.32*	0.40*	-		
SR	5.66	1.02	.86	0.46*	0.41*	0.55*	0.78*	-	
SS	4.95	1.00	.52	0.48*	0.53*	0.24*	0.27*	0.33*	-

*Notes*: SD standard deviation; α Cronbach’s alpha; PS “Perception of self”; PF “Planned future”; SC “Social competence”; FC “Family cohesion”; SR “Social resources”; SS “Structured style”.

* p = <0.01

Two models were analyzed; univariable logistic regression of all included items and a full model analyzing all items simultaneously (Table 3). Univariable analysis was conducted for all variables using logistic regression. However, regression command exlogistic was used for the variables “Loss of more than one child,” “Living alone but have a partner,” “Loss of child in 2013” or “2014”, which had a small sample size. Exact regression is an alternative to logistic regression, using the standard maximum-likelihood-based (CMLEs) logistic regression estimator, and it gives an inference that is more accurate in small samples. Maximum likelihood estimation used with the logit command can perform poorly in small sample sizes [36]. In the second model, the RSA was examined in two ways; first, with the covariates and the six resilience factors, which identify the unique contribution of each factor; and second; the covariates were included, but the six resilience factors were replaced by the total score on RSA (Table 3).

**Table 3:** Parental report on resilience factors as predictors of adjustment to grief in logistic regression analysis (confidence interval in brackets).

	Univariable logistic regression		Adjusted odds ratio (AOR)	
	OR (CI 95%)	*p*-value	OR (CI 95%)	*p*-value
Sex (men = 0, women = 1)	-.41 (-1.07-.25)	.222	.43 (.15–1.21)	.110
Marital status (married to the deceased child’s other parent = 0)				
Married to another than the deceased child’s other parent	1.39 (.49–3.95)	.539	1.89 (.38–9.52)	.439
Living alone, but have a partner	3.04 (.94–9.78)	.062	6.67 (1.26–35.35) .026*	
Single	.77 (.28–2.16)	.622	.46 (.08–2.61)	.382
How many children do you have	1.13 (.83–1.54)	.441	1.20 (.77–1.88)	.418
Loss of more than one child	1.15 (.09–10.39)	1.000	2.84 (.26–31.33)	.357
Loss of parent	.54 (.26–1.12)	.098	.30 (.10-.87)	.027*
Loss of parents-in-law	.57 (.23–1.37)	.209	.54 (.16–1.86)	.331
Years since loss of child (2009 = 0)	2.19 (.97–4.96)	.059		
2010	2.40 (.96–5.98)	.060	.65 (.14–3.02)	.581
2011	1.14 (.51–2.55)	.756	.16 (.03-.76)	.022*
2012	.62 (.29–1.34)	.225	.09 (.02-.41)	.002**
2013	.27 (.05–1.01)	.052	.02 (.00-.22)	.001**
2014	.50 (.09–2.07)	.471	.03 (.00-.29)	.002**
Perception of self	2.37 (1.58–3.56)	.000***	2.14 (1.05–4.38)	.036*
Planned future	1.94 (1.40–2.69)	.000***	1.41 (.84–2.38)	.194
Social competence	1.58 (1.15–2.18)	.005**	1.03 (.62–1.69)	.921
Family cohesion	1.12 (.84.1.51)	.439	.39 (.18-.82)	.013*
Social resources	1.82 (1.24.2.69)	.002**	3.96 (1.53–10.25) .005**	
Structured style	1.39 (.99–1.96)	.060	.62 (.35–1.09)	.098
Total score of RSA	2.71 (1.62–4.53)	.000***	3.42 (1.85–6.32)	.000***
Constant			.01 (.00-.38)	.014*
Observations			154	
Log likelihood			-65.0	
*χ*^*2*^			*χ*^*2*^_*19*_ 71.96	
*p*-value			.000***	
Pseudo R_2_			.35	

Note: Dependent variable: “Do you think you have come to terms with your grief?”

* *p* = < .05

** *p* = < .01

***p = < .000

## Results

The majority of the participants were women. Most participants were still married to the deceased child’s other parent, and more than half had relatively high education levels, having completed three or more years of college or university (Table 1). Table 1 gives an overview of the characteristics of the bereaved parents. Of the parents responding to the survey, most had lost a girl. At the time of death, the children’s median age was 14 years with a standard deviation of 6.81.

The Pearson correlation analysis of the six RSA factors is presented in Table 2 and includes the means, standard deviations, and coefficient alphas (α). Cronbach’s alpha showed sufficient internal consistency for all factors except *“Structured style”*, which had a low alpha of .52. The six RSA factors had significant positive correlations. The highest correlation was found between the two factors indicating intrapersonal resources (*“Family cohesion”* and *“Social resources”)*.

Table 3 presents the logit odds ratios (OR) with its confidence interval (CI) and the corresponding p-values. The log likelihood ratio Chi-square test statistics for the full model LR *χ*^*2*^_(19)_ = 71.96, *p* < .001 indicated that overall the model with all predictors was significant.

Regarding the univariable analysis (Table 3), all resilience factors showed a positive relationship with the dependent variable, and all were significant except for *“Family cohesion”* and *“Structured style*.*”*

In the multivariate analysis, the sex variable had an OR below 1, indicating that fathers had reported coming to terms with their grief to a greater extent than mothers had, but the result was not significant. The result for the variable “Living alone but have a partner” indicated that the odds of having come to terms with their grief was significantly increased. The loss of a parent predicted a decrease in having come to terms with their loss. Regarding the variable “Years since loss,” there were no significant differences between 2009 and 2010. However, for 2011 through 2014, there was a decrease in the odds of having come to terms with their loss when holding the other predictors constant. The results for these years were all significant at the .05 level (Table 3).

The total score of RSA was positively associated with a healthy grief outcome among the parents. The factors *“Perception of self”* and *“Social resources”* significantly predicted an increase in the odds of the respondent having come to terms with their grief. Regarding the factor *“Family cohesion”*, the result significantly predicted a decrease in the odds of a respondent having come to terms with their loss (Table 3).

### Fit statistics

The Linktest showed a significant value of _hat (p = .000) and a not significant _hatsq (p = .146). The Hosmer-Lemeshow test showed; χ^2^_(8)_ = 9.84; groups 10; p = .276, indicating that the variables included in the model fit the data well. There were high correlations between the factors *“Perception of self”* and *“Planned future”* and between *“Family cohesion”* and *“Social resources”* (Table 2), therefor the assumptions for collinearity were investigated. However, the variance inflation factor (VIF) was below the cutoff of 5.00 [37], where the highest value was found for factor five (*“Social resources”*) of 3.78 (mean VIF for AOR 1.80). All factors had 1/VIF above 0.2 with the lowest value of 0.264 for factor five, indicating no problems associated with collinearity in the data [37].

## Discussion

The aim of the present study was to explore whether resilience factors among cancer-bereaved parents predicted a healthy adjustment to the loss of their child. Overall, the total RSA score had a positive and significant association with the extent to which a parent had come to terms with their grief. Furthermore, three of the six RSA factors contributed significantly in predicting whether or not the parents in the present study replied that they had come to terms with their grief 2–8 years after they lost their child; specifically, the factors *“Perception of self,”* *“Social resources”*, and *“Family cohesion”* contributed.

The resilience factor *“Perception of self”* was positively associated with the degree to which the parents indicated that they had come to terms with their grief. The factor comprises a person’s ability to solve problems and have a positive view of the future. The findings in our study are supported by several studies [38–40] in which parents had indicated that after the loss they needed to reconstruct their identity and the meaning of life due to changes in the family. In a study by Stevenson et al. [40], parents emphasized the importance of internal resources as most helpful during the first year after the loss. Alam and colleagues [10] found in their study that at 18 months post-loss parents seemed to have had a personal growth which made it possible for them to move forward with new purpose in their lives. According to the present study, the parents’ ability to handle a stressful life event is an important resilience factor in order for them to be able to have a healthy adjustment to grief.

To have *“Social resources”* outside the family was an essential factor in predicting whether parents’ had come to terms with their grief. Social support such as friends, support groups and religious communities have been shown in other studies [28,41] to also be a significant source of support for bereaved parents. Some parents found comfort in helping other bereaved parents, and in some cases, helping others was a way to self-help [41]. In resolving parental grief, it was shown in different studies to be beneficial for parents to talk about their child and circumstances around bereavement experiences in a safe and nonjudgmental environment [42]. These findings are also in accordance with the DPM, which emphasizes the need for taking time to engage in social activities outside the family, such as being with friends, other bereaved parents, coworkers or health care professionals [40]. On the other hand, some parents indicated that it is not always easy for them to talk freely, and that in some circumstances there is a lack of understanding [40] or an uneasiness with family or friends when talking about the dead child [40,41].

Family cohesion is usually seen as an important protective factor in times of adversity, but enmeshed family bonds do not necessarily act as a protective factor. Interestingly, *“Family cohesion”* in the present study had a negative and significant association with the parents’ degree of having processed their grief and can therefore be considered a vulnerability factor. The findings in this study can indicate that the loss and grief reactions among parents of the deceased child are the “one thing” that they have in common in the post-loss period. They may be so preoccupied with talking about the deceased child and the circumstances surrounding the child’s illness and death that it can become overwhelming. Another interesting finding from the present study, which may confirm the assumption of family strains, is that parents who indicate that they have a partner but do not live with them, seems to have come to better terms with their loss compared to parents still living with the deceased child’s other parent. Through the lens of the DPM, relational problems can be seen as a family entrenched with a loss-orientated focus, unable to distance themselves from the loss and take “time-out” from their grieving process. According to the DPM the oscillation between loss- and restoration is an important feature in coming to terms with the loss [15]. A family environment adjusting to the loss of a child involves relational factors and not merely the individuals’ own grief reactions [43]. In some studies, it has been found that parents had experienced strain in their marriage and found communication with their spouse difficult [27,41]. This could be due to sex differences as well as having the marriage “put on hold” during the child’s illness and treatment period [44]. However, one study found that bereaved parents viewed their spouse as a source of support [45]. The contradictory findings from empirical studies may indicate that some couples are struggling in their relationship, whereas others find support and comfort with the other parent of the deceased child. Most research concerning sex differences in relation to grief processes are related to the loss of a spouse, whereas grief reactions regarding the loss of a child are scarcer [27]. In the present study, there were no significant differences between mothers and fathers regarding how they reported coming to terms with their grief.

As anticipated, “time since loss” had an impact on whether the parents experienced having come to terms with the loss of their child. There were no significant results between 2009 and 2010, but the results indicated that there appears to be a decline in grief reactions approximately six years after the loss, as these years predicted negatively and significantly that the parents had not completely processed their grief. Several studies have been conducted regarding the duration of parental grief reactions, but the findings are divergent. Vance and colleagues found in their study that grief reactions were reduced at eight months [46], whereas other studies have found that it takes a year or more [3,47,48] for parents to get their child’s death “into perspective and get on with their lives” [49]. Other studies have found that grief does not vanish, but that parents find a way to learn how to live with the loss [9], and therefore it is not possible to set a time frame for their grief reactions [9,50]. Because of the intimate relationship between parents and their children, grief is more intense and persistent than other types of losses [7], and the time course of grieving is uncertain and has great variability among bereaved parents [39].

In our study, parents experienced a negative association in coming to terms with the loss of their child when it was also combined with the loss of a parent or a parent-in-law. There are contrary views among researchers regarding exposure to several negative life-events, According to Masten [16], there is a greater risk of a poor outcome when there is an accumulation of adverse life events. However, Rutter ([51] p. 2) states that some individuals who are exposed to several adverse events seem to be more resistant to later adversities, exhibiting a “so-called steeling effect”. In the present study, multiple losses had a negative association with adjustment to grief.

It should be mentioned that the present study, including the articles referred to, is conducted in high-income countries (HICs). Randomized clinical trials have verified evidence-based treatments (EBT) for psychological distress in low- and middle-income countries (LMICs) with positive outcomes [52]. However, even though organizations like World Health Organization (WHO) support such interventions, few LMICs have been able to “scale-up or sustain” EBT because of poorly trained professionals, funding, logistics, etc. [52,53]. A report from WHO [54] claims that in LMICs there is a lack of mental health resources, the resources are disproportionately distributed and the uses are insufficient. No research regarding cancer-bereaved parents’ grief trajectories and level of resilience was found. This may have significant implications for a great proportion of bereaved parents since the survival rate of childhood cancer can be as low as 20% in LMICs [55] compared with Western Europe [56] and the United states [57] which have an approximately 80% survival rate.

Our findings partially provided support for resilience factors having a function as a buffer against adverse life events. According to Masten [16], resilience can be promoted by an enhancement of assets available and a facilitation of protective factors, as well as a focus on the reduction of risk factors and problems. Sandler and colleagues ([58], pp. 68–69) claim that bereaved individuals are “active agents” with their own experiences, skills, and needs, and that they can benefit from information and social support in order to discover their strengths and find their way through a difficult time. In our study, a considerable number of parents indicated that it took several years for them to adjust to the loss of their child. Health promotion among bereaved parents could possibly have clinical implications since a prolonged grief reaction has been shown to have psychological [3] and physical [4,5] consequences compared with a non-bereaved control group [3,59]. Goodenough and colleagues [60] found in their study that follow-up of cancer-bereaved parents in some cases was needed for several years following the loss due to a significant level of psychological distress. Several approaches to interventions can be suggested based on our findings. Identifying individuals that might have a low score on resilience can enable health-care professionals to recognize parents in risk of developing poor health or prolonged grief. Results from our study indicated that couples who experienced strain in their relationships or had experienced multiple losses also indicated that they not had worked through their grief. Interventions such as grief counseling can be an approach to help couples with marital strain [16], either individually or as a couple. Grief counselling offers the opportunity to both identify problems regarding the child’s death and discover other issues with which couples may struggle [61]. Less cohesive couples may also need to be encouraged to participate [61] because parents’ reactions to the loss can affect each other [62]. Family therapy can help the family to find existing resilient personal resources and to use the resources in a way that strengthens the family cohesion, and helps them use their strength to move forward with their lives [63]. In a study by Kreicbergs and colleagues [64] evidence was found that psychological support from health-care professionals during the last month of their child’s life had a positive impact on the parents’ long-term grief outcome.

Iacoviello and Charney [65] recommend using role models to enhance resilience among individuals who are experiencing a difficult time. A role model could be someone who has had similar experiences and thereby can be a resourceful confidante. Support groups from the treating hospital or the Children’s Cancer Society can be such role models who can help parents realize that even if they are going through a difficult time, it is possible to live through the adversities and still have the opportunity to have a good life.

Nurturing aspects that already have a positive influence on parents’ grief reactions can also be constructive. In our study, having social resources had a positive impact on parents’ ability to work through their grief. Health-care professionals can encourage the parents to foster their relationship with family and friends during the illness and following the loss. Social support networks can be the difference between a resilient outcome and psychopathology [65]. Further, emotional support can influence an individual’s perception of himself, and thereby strengthen intrapersonal resilience traits such as optimism, self-efficacy, and coping skills [65]. Investigating parents’ social networks can also discover families with limited access to support. Health-care professionals can then inspire the parents to participate in support groups for bereaved parents, or perhaps help them to contact their general practitioner to ensure some degree of follow-up after the loss. In a theoretical model Snallow and Paul [66] recommended that health promotion involving health-care professionals in the parents’ home communities can focus on developing skills, enhancing coping and resilience among bereaved individuals, and minimizing harmful factors.

Today there exists no consensus on a model or guide for a resilience training program [67]. However, a review discovered several studies with promising results regarding interventions to improve resilience and mental health in adult populations exposed to adversities [67]. The review revealed studies aiming at promoting resilience such as optimism, self-efficacy, positive attributional style, and social support after exposure to stressors was efficient ([67], p. 27). However, the studies in this review did not include studies of cancer-bereaved parents, which subsequently provides a direction for future research. Another recommendation is to conduct longitudinal studies focusing on interventions aiming at strengthening parents’ resilience to investigate whether interventions are efficient and if parents’ adaptation to loss changes over time.

## Strength and limitations

A strength of the present study is the novelty of using a resilience scale as a predictor of the grief outcome of parents following the loss of a child to cancer. Another strength is the high internal response rate of 95.7% for the variables included in the present study. Among the limitations is the cross-sectional design, which limits the possibility of assessing whether the parents’ resilience traits contribute to a healthy grief adjustment over time. Another limitation is the self-selection of the study design, which may affect who chose to participate in a survey, and hence, it is difficult to generalize the findings to a broader population of cancer-bereaved parents. Yet, another limitation is that for the dependent variable, two of the response alternatives were merged due to the low proportion of participants, indicating that they have not come to terms with their grief. It is possible that there are some special characteristics of those participants who answered that they have not come to terms with their grief at all; hence, that information is lost when merging the two response alternatives. The retrospective design of the study is another limitation, which may have caused recall-bias in the data materials. The low response rate is another limitation and therefore, the results should be treated with caution in reference to generalization.

## Conclusion

The present study shows evidence for resilience factors as significant predictors for the healthy adjustment to grief. However, the results also indicate that there could be tension in the relationships of bereaved parents. Health-care professionals should be aware of the potential risk that some parents can have and assess the need that individuals may have for professional follow-up in order to strengthen the parents’ resilience. The results also showed the importance for health-care professionals of gathering information about additional risk factors that families could have, as the results showed that the loss of a parent or a parent-in-law was also a risk factor in the present study.

### Ethics

The Regional Committee for Medical and Health Research Ethics (Ref. 2014/1997/REK Midt) approved the study.
